# Formation of Black Silicon in a Process of Plasma Etching with Passivation in a SF_6_/O_2_ Gas Mixture

**DOI:** 10.3390/nano14110945

**Published:** 2024-05-28

**Authors:** Andrey Miakonkikh, Vitaly Kuzmenko

**Affiliations:** Valiev Institute of Physics and Technology of RAS, Nakhimovsky av. 34, 117218 Moscow, Russia; kuzmenko@ftian.ru

**Keywords:** black silicon, reactive ion etching, plasma etching, passivation, plasma oxidation, anisotropic etching, light absorbance, reflectivity

## Abstract

This article discusses a method for forming black silicon using plasma etching at a sample temperature range from −20 °C to +20 °C in a mixture of oxygen and sulfur hexafluoride. The surface morphology of the resulting structures, the autocorrelation function of surface features, and reflectivity were studied depending on the process parameters—the composition of the plasma mixture, temperature and other discharge parameters (radical concentrations). The relationship between these parameters and the concentrations of oxygen and fluorine radicals in plasma is shown. A novel approach has been studied to reduce the reflectance using conformal bilayer dielectric coatings deposited by atomic layer deposition. The reflectivity of the resulting black silicon was studied in a wide spectral range from 400 to 900 nm. As a result of the research, technologies for creating black silicon on silicon wafers with a diameter of 200 mm have been proposed, and the structure formation process takes no more than 5 min. The resulting structures are an example of the self-formation of nanostructures due to anisotropic etching in a gas discharge plasma. This material has high mechanical, chemical and thermal stability and can be used as an antireflective coating, in structures requiring a developed surface—photovoltaics, supercapacitors, catalysts, and antibacterial surfaces.

## 1. Introduction

Black silicon is a synthetic nanomaterial that contains high-aspect-ratio nanoprotrusions or nanoholes on its surface, usually produced by etching of the planar silicon surface. Many of these structures on SEM images resemble grass, and so have received the name nanograss. It is possible to judge the formation of developed nanostructures on a sample even without the use of instruments: a shiny metal-like silicon surface acquires a velvety dark or even black color due to significant plasmon absorption of light.

This material has high mechanical, chemical and thermal stability and can be used in a wide range of applications [1]. The most prominent of them is antireflective coatings [2,3,4,5]. The other applications use black silicon in structures requiring a developed surface—photovoltaics [6,7], photodetection [8], supercapacitors [9,10], catalysts [11,12,13], gas sensors [14], and THz-range radiation emitters [15]. The recently discovered antibacterial properties of such surfaces are also of interest and have led to significant efforts to identify the mechanisms of this phenomenon [16,17,18]. The reflectance of black silicon is between 20 and 2%. In terms of performance, the best examples of black silicon are significantly superior to other antireflection materials.

Obviously, the main challenge for the commercial production of black silicon devices and materials is to find ways to produce it cost-effectively. Here, the use of self-formation technology is promising, when structures are created as a result of self-organization processes without the use of nanoscale lithography. The most interesting examples of self-formation of nanostructures are observed in plasma processes of various natures. For example, in arc discharges, it is possible to observe not only the formation of nanostructures but also phase transformations, such as their crystallization [19,20,21].

For black silicon, several cost-effective formation technologies are known [22]: inductive coupled plasma reactive ion etching (ICP RIE, or short ICP) process in an atmosphere of SF_6_ and passivation gas [23], metal-assisted wet chemical etching (MACE) process based on Ag or Au catalyst particles in aqueous solutions of HF and H_2_O_2_ [24], electrochemical etch cell used for macro-porous silicon [25], plasma-less gaseous etching by fluorine [26], and laser irradiation (texturing) [27]. The properties of structures formed by different methods differ radically in terms of feature dimensions, topology, lifetime of carriers and crystallinity.

Although the MACE process is one of the most cost-effective, the disadvantage of this process is the metal contamination of silicon, and, as a result, a reduction in minority carrier lifetime in silicon, especially in p-type silicon [22]. The laser irradiation method of black silicon production makes it possible to achieve absorption in a wide spectral range, but it is very expensive and incompatible with mass production. The RIE method can be used both with and without a mask. Using a mask makes it possible to obtain ordered periodic structures with defined geometry, for example honeycomb [22], but this requires an additional technological procedure (lithography), which complicates production. Maskless methods for forming silicon are based on self-organization of structures and can be implemented in plasma or liquid.

In this article, we focus on plasma methods for the formation of black silicon based on the etching of single-crystalline silicon in the plasma of fluorine-containing gases, with the most effective sources of dense low-temperature low-pressure plasma, for example ICP tools, the widespread development of which is associated with advances in microelectronics technology.

This maskless method has high productivity and scalability, and unlike RIE methods using polymer passivation, it leaves the silicon surface uncontaminated (the thickness of the silicon oxide formed during plasma oxidation is close to the thickness of the native oxide formed on silicon in an air atmosphere). The advantage of the maskless process is that it can be carried out without the use of lithography, in one stage.

Currently, commercially available plasma etching tools allow processing silicon wafers with a diameter of up to 300 mm (examples up to 450 mm are known). Typical plasma parameters are as follows: pressure 1–20 mTorr, electron temperature approximately a few eV, a density of charged particles 10^10^–10^12^ cm^−3^. Such parameters ensure the etching of silicon and other microelectronic materials at rates up to several microns/min, making it possible to reduce the number of electrically active defects in the structure of semiconductors and dielectrics subjected to processing and, if necessary, the required degree of anisotropy.

For silicon, the problem of etching anisotropy is particularly important—when etching in a fluorine-containing plasma, spontaneous etching of silicon occurs by atomic fluorine radicals, which, although they provide a high rate of etching of microstructures, do not allow to obtain a vertical profile of the side wall of the etching when using a lithographic mask to create structures with the required lateral dimensions.

The most known deep silicon etching processes: the cyclic original Bosch process [28] and a lot of its modifications, the continuous cryogenic etch process [29], the cyclic STiGer process [30], and continuous pseudo-Bosch process [31].

All these processes are designed with fluorine chemistry of SF_6_ based plasma that provides the fastest etch of silicon enhanced by ion bombardment from plasma. The differences are in the approach used to suppress the isotropic etching of the structure sidewalls to achieve required anisotropy and of etch process. The Bosch process consisting of sequential alternating steps of etching and polymer sidewalls passivation gives a typically scalloping profile and polymer contamination of sidewalls. The SF_6_ plasma step etches the silicon, and the C_4_F_8_ plasma step deposits protection layer. Otherwise, the cryogenic processes utilize Si_x_F_y_O_z_ passivation layer stable only at cryogenic temperature (<−80 °C). First version is continuous regime with SF_6_+O_2_ plasma [29], and the second one is gas chopping process SiF_4_+O_2_/SF_6_ which is patented as STiGer etch process [30]. The first gives minimal sidewall roughness (<5 nm) with control of profile angle, and the second is faster, also clean but gives scalloping profile similar Bosch process. In pseudo-Bosch process (or mix-process) etching is performed in a mix of SF_6_ and C_4_F_8_ plasma. By changing the ratio SF_6_:C_4_F_8_, the sidewall profile angle can be controlled. Increasing the ratio improves the etch rate but reduces the selectivity and decrease profile angle. Increasing the plasma power again reduces the selectivity with a slight improvement in etch rates. This process is characterized by contamination of the walls with difficult-to-remove polymers, but provides very high resolution and is not prone to scalloping.

There is only one type of process that does not involve the use of polymer-forming plasma and cryogenic temperatures. This is a cyclic oxidation-etching process in which oxygen plasma is replaced by SF_6_ plasma cyclically. Active oxygen radicals lead to the formation of a stable oxide film on the side walls, and fluorine radicals, when stimulated by vertically incident ions, effectively etch the bottom of the structure. The result is a well anisotropic structure. This process was proposed in the work [32] and patented [33]. The independent development of this process under the name CORE is also known in works [34,35], in which, among other things, a method for the formation of black silicon was proposed. The disadvantage of this method is that it is used in CCP mode, which limits the etch rate.

In all these processes, self-formation of black silicon is possible. The phenomenon of self-formation of micro- and nanostructures on the silicon surface during plasma etching has been studied experimentally and by modeling [36,37] in various technological processes based on fluorocarbon film deposition during the etching. The most typical form of nanostructures are needles of various shapes and sizes.

In addition to the deposition of fluorocarbon films, another approach to passivation of side walls during Si etching is oxidation of the surface in plasma under the influence of oxygen radicals [38,39]. When interacting with the Si surface, O radicals can form SiO_2_, which is stable at room temperature. This plasma oxidation process is in saturating mode, since when a SiO_2_ layer is formed on the surface, the transport of radicals from the plasma to the Si layer is blocked. At the same time, fluorine radicals also do not interact with the silicon surface. The difference between the etching mechanism of silicon oxide and the etching of silicon in a fluorine-containing plasma is that silicon is spontaneously etched by fluorine radicals, and silicon oxide is practically not subject to spontaneous etching without the presence of a fluorine-enriched polymer film and ion bombardment. In addition, the rate of linear etching of silicon is many times higher than the rate of etching of silicon oxide. This opens up the mechanism for the formation of side wall passivation and the implementation of anisotropic etching. In [39], the possibility of anisotropic etching of silicon in a reactor with a CCP source in SF_6_/O_2_ plasma with an O_2_ fraction of 50% was demonstrated.

At the same time, excessive passivation (which is observed with an increase in the proportion of oxygen in the plasma or a decrease in temperature) can lead to a stop even of directional etching, in the case when the bombardment of ions is not enough to constantly sputter the oxidized layer on horizontal etching surfaces. In the intermediate regime, when passivation on horizontal surfaces is quite stable but individual stochastic defects can form and be etched, the formation of black silicon is possible. Apparently this is facilitated by the fact that due to the higher sticking coefficient of oxygen, it is much more difficult for it to penetrate into the formed deep structures than fluorine.

**Figure 1:**
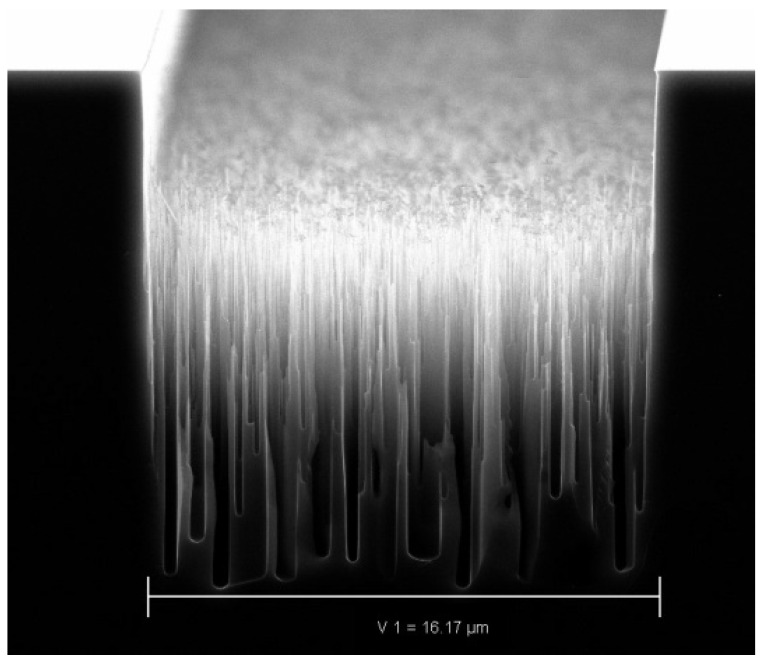
Example of silicon nanograss formation in continuous cryogenic deep silicon etching process in mix of SF_6_ and O_2_ with over-passivation.

The results of modeling the formation of nanograss in a continuous cryogenic process of deep etching of silicon in the plasma of a mixture of SF_6_ and O_2_ (see Figure 1) are presented in the article [40]. The idea of forming nanoroughness on the side wall of the etching structure was also proposed there. A randomly generated depression on the surface changes the conditions for the transport of particles to its bottom. As a result, more particles that stimulate etching—fluorine radicals—get to the bottom than particles that passivate it—oxygen radicals. In these conditions, the accidentally created depression continues to etch faster. On the other hand, the tips of the needles obtained by etching, on the contrary, are well passivated by oxygen, but are subject to less bombardment by ions as a result of local charging.

It is obvious that factors promoting passivation (an increase in the passivating content in the plasma, a decrease in temperature) will contribute to the formation of black silicon during etching. On the other hand, an increase in temperature and an increase in the ion flux density shift the balance towards classical etching. This idea was used in [41] to easily diagnose silicon etching anisotropy in an era when the availability of electron microscopes was still low. The presence of a region of technological parameters in which black silicon is formed suggests that on its boundary there is a region in which the etching is anisotropic with vertical walls. This approach was called the black silicon method and was fruitfully used in a number of works in different kind of etching conditions and plasma-forming gases.

The optical properties of nanostructured surfaces are widely studied theoretically and experimentally [42,43,44,45]. Methods for lowering the reflection coefficient of black silicon are also known from the literature. One of them is based on the conformal coating of the structure surface with a dielectric layer [2,44,46]. This approach can be implemented using the atomic layer deposition method. The advantages of this approach are determined by the high degree of uniformity of the films obtained by this method over the surface of the wafer, the high reproducibility of their optical characteristics, and their high conformality (the ability to cover both the side surfaces and the bottom of even high-aspect structures with a layer of the same thickness). The latter property was both studied experimentally and modeled [47,48].

Thus, the objectives of this article are as follows. The investigation of the formation of black silicon nanograss structures in the process of high-performance plasma etching. The composition of the plasma-forming mixture (sulfur hexafluoride—oxygen) varied over a wide range. Regularities have been established that connect the nature of the resulting structures both with the external parameters of the discharge and with the concentrations of fluorine and oxygen radicals in the discharge. In addition, the dependence of the resulting structures on the substrate temperature and bias voltage was also studied. Further, single- and double-layer ALD coatings of black silicon structures were implemented. For all listed structures, the spectral reflectance was studied.

## 2. Materials and Methods

### 2.1. Structures Manufacturing

The experiments were carried out in a Plasmalab plasma etching tool (OIPT, Bristol, UK), specialized for microelectronic production, in the plasma mix of SF_6_ and O_2_ gases, at sample temperatures from −20 °C to +20 °C and an electrical bias on the sample from 50 to 150 V. The input power in the discharge ICP was 1750 W (2 MHz), total flow of feeding gases was 50 sccm. The RF power (13.56 MHz) applied to sample chuck was 30 W (corresponding DC bias accelerating plasma ions was 150 ± 20 V) and 10 W (DC is 58 V) For the etching experiments, we used coupons of Si (100) wafers (p-type doped, with resistivity 10 Ohm∙cm, thickness 550 µm) with an average size of 5 × 5 cm. The coupons were glued by PFPE grease to a 100 mm silicon wafer used as a sample carrier. The He pressure up to 6–7 Torr were built during the etching under the carrier to provide good thermal contact with the water-cooled table.

### 2.2. Studying of Topology and Autocorrelation Function

The topology of the structures was studied using a cross-sectional images and top-view images obtained using the scanning electron microscope (Smart SEM Supra 55, Carl Zeiss AG, Jena, Germany).

**Figure 2:**
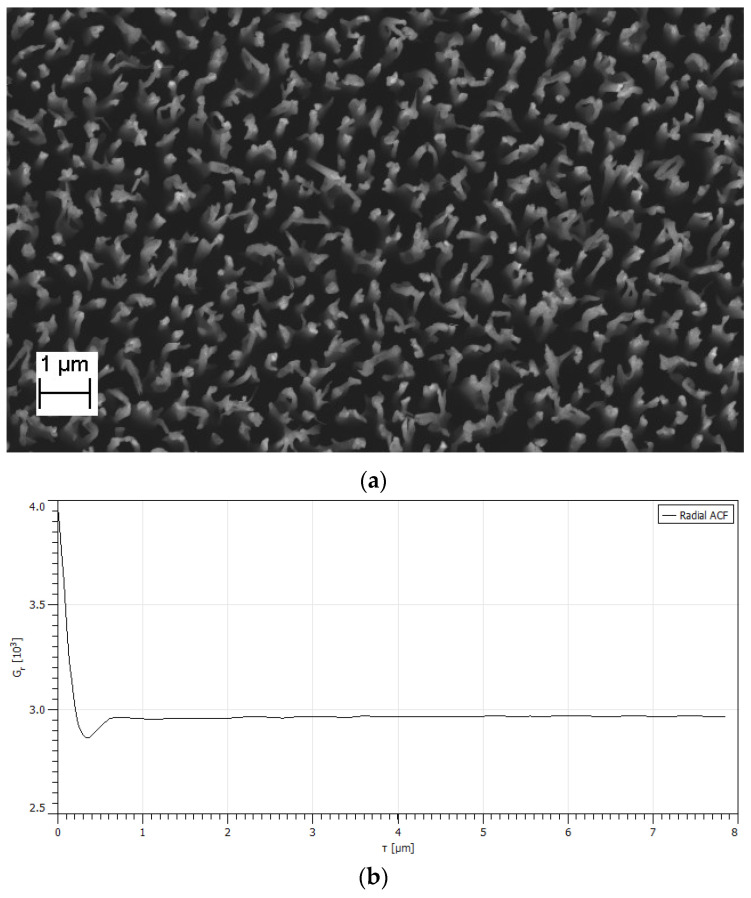
The example of autocorrelation function: (**a**) SEM picture of top view of black silicon; (**b**) radial autocorrelation function for that image.

Although, as will be shown below, the resulting structures are generally random and aperiodic in nature (see top view SEM image on Figure 2a), it can be noted that the characteristic distance between features varies depending on the selected characteristics of the structure manufacturing process. To characterize the quasi-period, the autocorrelation function is most suitable, which can be calculated directly from the top view image obtained using a scanning electron microscope. The two-dimensional (2-D) autocorrelation function (ACF) of an image statistically characterizes the spatial pattern within that image and presents a powerful tool for fabric analysis. We used Gwyddion program (v. 2.66)—a modular open-source software for scanning microscopy data processing [49].

The autocorrelation function is given by equation:

(1) $$G\left(\tau_{x},\tau_{y}\right)=\iint_{-\infty }^{+\infty }z_{1}z_{2}\cdot \omega \left(z_{1},z_{2},\tau_{x},\tau_{y}\right)dz_{1}dz_{2}=\underset{S\to \infty }{\lim}\frac{1}{S}\iint_{S}\xi (x_{1},y_{1})\cdot \xi (x_{1}+\tau_{x},y_{1}+\tau_{y})dx_{1}dy_{1}$$

where $z_{1}$, $z_{2}$ are the values of image brightness at points $\left(x_{1},y_{1}\right),\left(x_{2},y_{2}\right)$; $\tau_{x}=x_{1}-x_{2}$ and $\tau_{y}=y_{1}-y_{2}$. S in Formula (1) means that integration is carried out over the area, in the limit over the entire image. The function $\omega \left(z_{1},z_{2},\tau_{x},\tau_{y}\right)$ denotes the two-dimensional probability density of the random function ξ(x, y), corresponding to points $\left(x_{1},y_{1}\right),\left(x_{2},y_{2}\right)$, and the distance between these points τ.

On case of black silicon, we can also introduce the radial ACF G_r_(τ), i.e., angularly averaged two-dimensional ACF, which contains the same information as the one-dimensional ACF for isotropic surfaces:

(2) $$G_{r}\left(\tau \right)=\int_{0}^{2\pi }W(\tau \cos\varphi ,\;\tau \sin\varphi )d\varphi .$$

The position of the main minimum of this function determines the distance relative to the peak at which the appearance of other (closest) peaks is least likely; this is half the quasi-period of the structure or the autocorrelation length. Naturally, long-range order is not observed in such structures; for this reason, there is always one minimum for ACF. The ACF function can be calculated from any pixel image, including an image obtained on a scanning electron microscope, which is considered as a bitmap with the dependence of pixel brightness on a black-and-white scale depending on the coordinate. In this case, the integral in (2) is replaced by a sum with averaging. Figure 2b shows the radial autocorrelation function calculated for the image from Figure 2a. A typical picture is observed with one minimum at 0.35 μm and the absence of any signs of order at large distances. In future work, we will only give the numerical value at which ACF has a minimum.

### 2.3. Studying of the Reflectance

The reflectance of the surface with black silicon was studied using an Ocean Optics spectrometer with a resolution of approximately 0.35 nm in the range of 200–900 nm and a white tungsten light source (BIM-6210, Brolight, Hangzhou, China, 400–2000 nm). The measurements were carried out using an integrating sphere [50] (SIM-3003, Brolight, 30 mm in diameter with 6 mm aperture), which makes it possible to estimate not the specular reflection intensity, but also the diffuse component. Such measurements are important to estimate how much light is absorbed and how much is scattered, regardless of the direction of scattering. The integrating sphere has an internal coating with a high reflectance, which reaches 0.98–0.995 depending on the spectral region. The sample occupies a small (but finite and known part) of the inner surface and is illuminated with a beam of white light close to normal. The light for measuring the intensity on the spectrometer was selected from the sphere through a thin aperture. Multiple reflections of light inside the sphere are taken into account as the sum of a convergent series, which determines the sphere multilayer—a coefficient taken into account when interpreting the data. In our case, the setup was preliminarily calibrated using a single-crystalline silicon sample, the optical characteristics of which are well known [51], and the reflectance can be determined using Fresnel formulas.

### 2.4. Dielectric Coatings

In this work, to deposit a dielectric, we applied atomic layer deposition (ALD) [47] of hafnium and aluminum oxides on the surface of black silicon. For this, the organometallic precursors trimethylaluminum and tetraethylmethylaminohafnium, respectively, and oxygen plasma were used. The processes were performed on ALD tool FlexAl (OIPT, Bristol, UK) at the plasma enhanced deposition mode at 300 °C. The deposition processes were tested on blank silicon wafers and homogeneity and optical properties of films were measured by spectral ellipsometry [52] on Woollam M-2000X tool (Lincoln, NE, USA), using Cauchy model. The measured growth rate was 0.099 nm/cycle for HfO_2_ and 0.101 nm/cycle for Al_2_O_3_, with refractive indices at 632 nm equal to 2.01 and 1.65, respectively. The applied film thickness was 5–80 nm.

In our work, dielectric coatings of one and two layers were simulated. Since modeling on the black silicon surface is complicated by the need to accurately take into account the topology, this task is extremely complex (note that successful approaches to its solution were developed in [44]). In our work, as reference data, we take the calculation of the reflectance of planar silicon coated with one or two layers of dielectric materials, performed according to Fresnel formulas and taking into account reflections at interfaces and interference. The spectral dependences of the refractive indices of dielectric films determined by the method of spectral ellipsometry were taken for calculations.

### 2.5. Plasma Diagnostics

In order to identify the main mechanisms of the formation of black silicon, we carried out plasma diagnostics in order to determine the influence of particles such as oxygen radicals and fluorine radicals, it is necessary to directly measure their concentrations, since the behavior of these quantities in the plasma of such a mixture is very complex and nonlinear, determined by the variety of chemical reactions in plasma of complex composition [53,54].

The density of F and O radicals was determined by optical emission actinometry. In this method, the density of a plasma particle is calculated by comparing the optical emission intensity of the particle and that of an actinometer inert gas (in our case Ar) with a known density. In our experiments, the Ar fraction in the plasma was 7–10%. The fluorine radical density was determined using F 685.6 nm/Ar 750.4 nm and O 777.4 nm/Ar 750.4 nm actinometrical pairs. The emission spectra were measured using an HR4Pro spectrometer (Ocean Insight, Orlando, FL, USA) with a spectral resolution of 0.35 nm. We used the calibration from the spectrometer manufacturer for line intensities and wavelengths. The optical spectra were normalized to the fiber transmission function. The detailed approach applicable for low-pressure ICP discharge is described in [55].

Langmuir probe diagnostics is a useful method for determination of plasma charged-particles related properties, which is important for kinetics understanding and radical concentration calculation. Measurements of I–V curves were made with ESPion Advanced probe (Hiden Analytical, Warrington, UK) in a range of −100–+20 V with 0.1 V step. The cylindrical tungsten probe was 10 mm in length and 0.3 mm in diameter. The measuring probe is mounted on a long (~200 mm) dielectric holder, which makes it possible to carry out measurements in the central region of the discharge. In order to prevent any kind of contamination on the probe surface, between the measurements, the probe was maintained at a potential of 15 V and was cleaned with an ion bombardment, which allows obtaining undisturbed probe characteristics [56,57]. Probe size measurements before and after plasma diagnostics prove invariability of feature size (no probe material etching takes place during measurements). The electron temperature, plasma potential and electron and positive ion concentration were calculated from the I–V characteristics. The ion current was determined using orbital-motion-limited (OML) theory [58]. The ion current branch of I–V characteristic was approximated by the expression I~V^α^. The deviation of α from 1/2, predicted by OML theory, was less than 20%, which indicates the validity of applying OML theory. Subtracting the ion current from I–V characteristics gives the electron current. The second derivative of electron current over potential is proportional to the electron energy distribution function and linear dependence of the natural logarithms of electron current with the probe potential corresponds to Maxwellian electron energy distribution. The electron temperature was calculated by the slope of the natural logarithm of electron current versus probe potential.

## 3. Results

**Figure 3:**
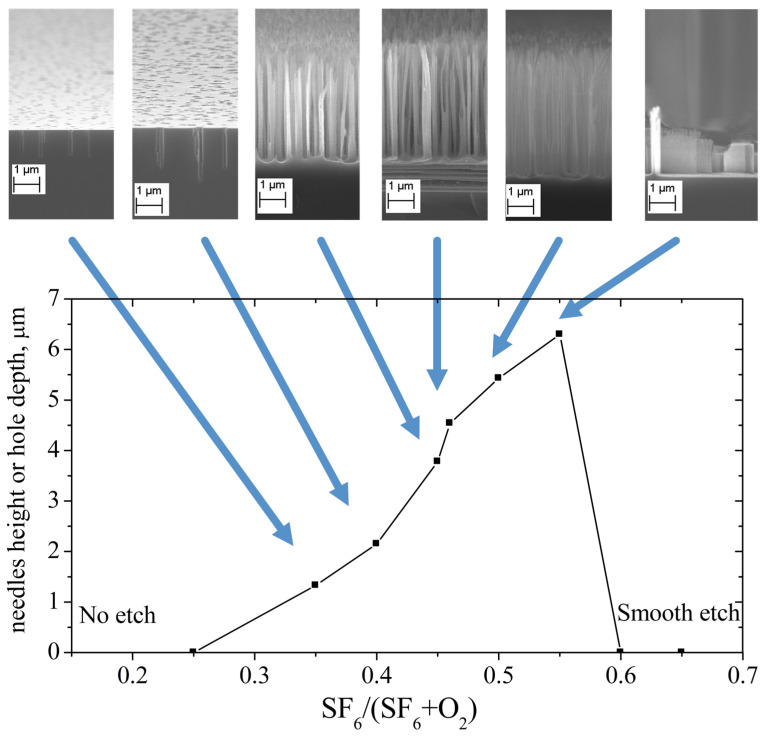
The needles height or holes depth over SF_6_ fraction in plasma mixture, duration of etch is 300 s, temperature is −20 °C.

In order to identify the patterns of formation of structures during etching, first of all, an experiment was carried out with etching of structures for the same time equal to 300 s at the same temperature −20 °C. In this case, the composition of the mixture changed so that the proportion of SF_6_ changed from 0.25 to 0.75 at maintaining a constant flow of gases into the reactor. Using an electron scanning microscope, the depth of the structures was measured: if we observed individual peaks, their height, if individual holes were observed, their depth. Figure 3 shows a graph of the observed etch depth as a function of the composition of the plasma-forming mixture. The insets show microimages of a cross-section of the silicon surface after etching. It can be seen that at SF_6_ fluxes less than 0.25, no surface development is observed. It would be correct to describe this result with the fact that etching does not occur at all, and the surface is passivated by an oxide film. Typically, when etching in a fluorine-containing plasma, the selectivity of silicon etching with respect to the oxide is 100:1. When the SF_6_ content increases to 0.4, individual thin holes are formed on the surface, the number of which increases with increasing SF_6_ content in the mixture. The mechanism of their formation was discussed above; if, as a result of random events (for example, several ions hitting one center), local destruction of the oxide film occurs, then etching continues at the bottom of the formed hole, and the transport of the passivation particles Is hindered.

When the SF_6_ fraction increases above 0.45, the holes merge and lonely columns remain. The horizontal dimensions of the columns decrease, the etching depth increases, and the distance between them increases. When the SF_6_ fraction reaches 0.55, only individual columns are observed on the surface, and the distance between them exceeds several microns. When the SF_6_ fraction is more than 0.6, no structures are observed on the surface; moreover, there is no roughness even visible in a scanning electron microscope. Although formally the etching depth in this case is estimated as zero, it should be noted that the entire surface is etched as a whole at a fairly high rate, and the etching is of a polishing nature, that is, even random structures disappear.

It is reasonable to believe that the observed pattern in Figure 3 shifts to the left or right as other factors affecting the kinetics of passivation and etching change.

**Figure 4:**
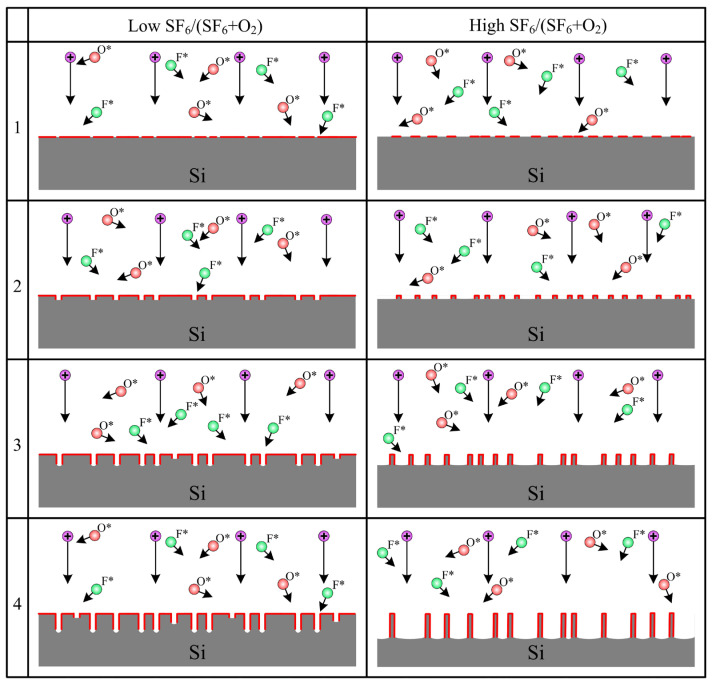
Schematic representation of the formation of black silicon at high oxygen content in the feed mixture (**left**) and low oxygen content in the feed mixture (**right**). In this figure, positive ions are shown as circles with a plus sign. F* and O* stand for fluorine and oxygen radicals, respectively.

Figure 4 schematically summarizes the patterns of black silicon structures presented above (Figure 3) and clarify the mechanism of black silicon formation discussed in the introduction at different oxygen contents in the plasma-forming mixture. At a high oxygen content (Figure 4, left), at the initial stages of etching, the silicon surface is effectively oxidized; however, a sufficient fluorine content and the presence of ion bombardment can lead to random destruction of the oxide mask in certain spots. The etching of opened silicon occurs at a higher rate than the etching of silicon oxide, leading to the formation of individual narrow holes. The walls of these holes are vertical, since even a small amount of oxygen in the gas mixture suppresses spontaneous etching of walls. It should be noted that with increasing etching time, the holes can expand due to slow lateral etching, thereby increasing the autocorrelation distance (minimum ACF).

On the other hand, with a low fraction of oxygen in the mixture (Figure 4 on the right), most of the silicon surface in the initial stage of etching is not passivated, and a sufficient thickness of the oxide layer is achieved only on a small part of the surface. This leads to the formation of protruding bumps. Electric field effects associated with the charging of their surface deflect ions from these formations, inhibiting etching in these areas. This leads to the formation of needles, the side walls of which are well passivated due to spontaneous oxidation by oxygen radicals. It should be noted that with increasing etching time, individual needles can die due to lateral etching of the surface, thereby increasing the autocorrelation distance (minimum ACF).

**Figure 5:**
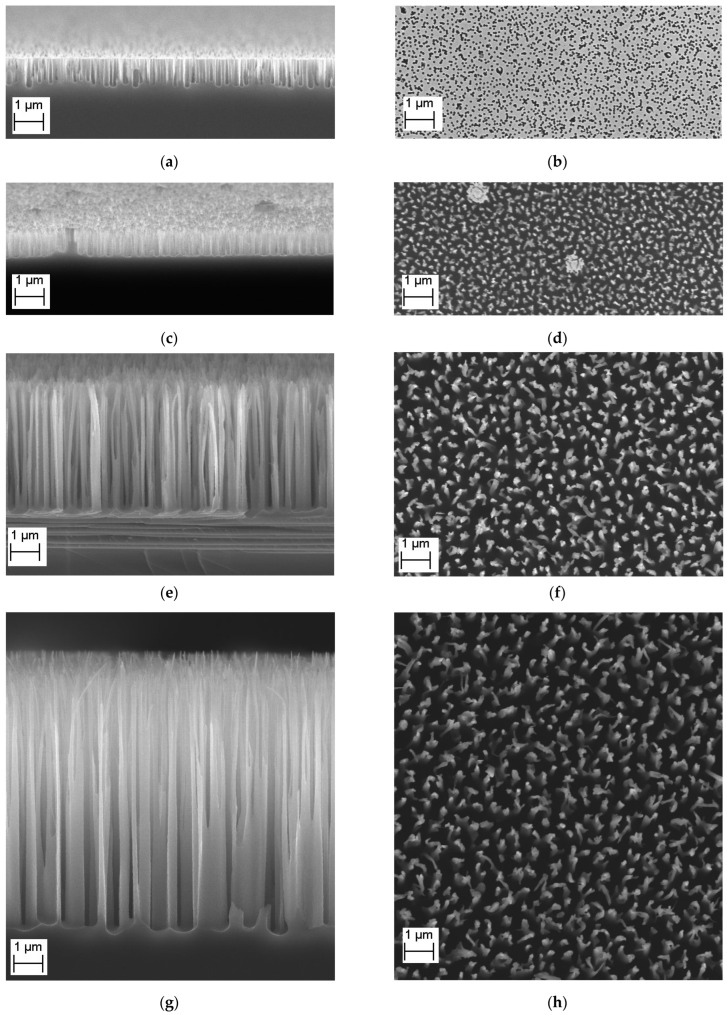
SEM pictures of black silicon for different duration of etching ((**a**,**c**,**e**,**g**)—side view, (**b**,**d**,**f**,**h**)—top view): (**a**,**b**) 60 s; (**c**,**d**) 120 s; (**e**,**f**) 300 s; (**g**,**h**) 600 s. For all processes the same set of parameters was chosen—temperature is −20 °C, fraction of SF_6_ is 0.46.

Figure 5 shows the temporal evolution of the etching pattern from smooth silicon over time. The same parameters were used for all samples—temperature is −20 °C, fraction of SF_6_ is 0.46.

On the left are images of structures in a cross-section, perpendicular to the surface, on the right is a top view. The scale of the drawings is the same. It can be seen that when structures are formed, their depth does not increase immediately. At the first stage (Figure 5a,b), small holes are formed, which then (Figure 5c,d) merge, forming nanocolumns; over time, the columns become rarer (Figure 5e,f), that is, with prolonged etching, some of them are randomly destroyed, and the tops of the remaining the columns become sharper (Figure 5g,h).

Figure 5 gives an idea of what the surface of the samples looks like to the human eye (images are not shown because they are not very well conveyed when photographed). It can be seen that the image with the shortest etching time of 60 s is closest in appearance to crystalline polished silicon. The specular reflection from it is practically undisturbed, since the defects are much smaller than the wavelength. The color of this sample is slightly darker than silicon. A sample with an etching time of 120 s, although the specular reflection is worse, still does not create the impression of a black velvet surface; samples with 300 s and 600 s look black, and the second is much darker.

**Figure 6:**
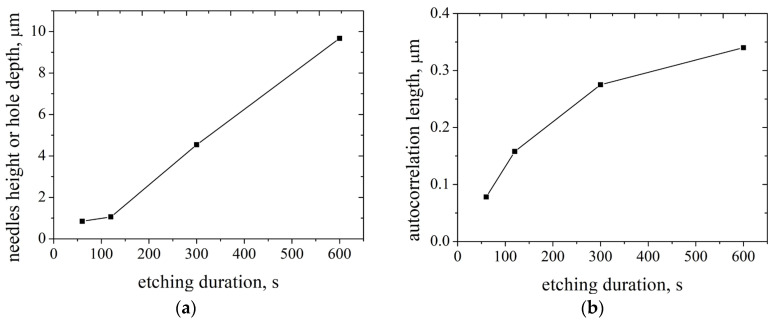
Black silicon characteristics over etching duration: (**a**) needles height or holes depth; (**b**) autocorrelation length. For all processes the same set of parameters was chosen—temperature is −20 °C, fraction of SF_6_ is 0.46.

Numerically performed observations are illustrated in Figure 6. Figure 6a shows an increase in the etching depth with etching time, in which a dependence, although close to linear, is shifted relative to zero. The autocorrelation length increases monotonically with increasing etching duration, since as individual columns disappear, the distance between them increases. It should be noted that the autocorrelation length is growing with saturation—as the distance between the columns increases, they have less influence on each other through the reflection and separation of energetic plasma particles.

**Figure 7:**
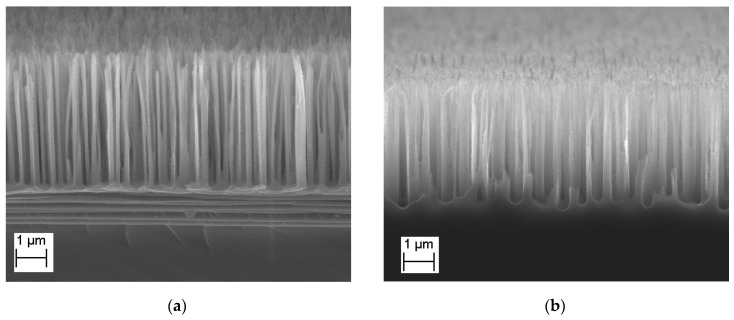
SEM picture of black silicon for different DC bias voltage during etching process: (**a**) 160 V; (**b**) 58 V. For both processes the same set of parameters was chosen—temperature is −20 °C, fraction of SF_6_ is 0.46.

The morphology of structures obtained with different bias voltages (corresponding to the energy of plasma ions reaching the surface and moving in the vertical direction) equal to 160 V (Figure 7a) and 58 V (Figure 7b) is generally similar. For structures obtained with a lower bias voltage, the cores have a slightly smaller etching depth (4.0 µm compared to 4.5 µm), a more rounded bottom shape, and a shorter autocorrelation length (0.23 µm compared to 0.28 µm). This can be explained by less efficient sputtering of the passivating oxide film at the bottom (sputtering threshold is approximately 40 eV) and less reflection, which leads to lateral etching.

**Figure 8:**
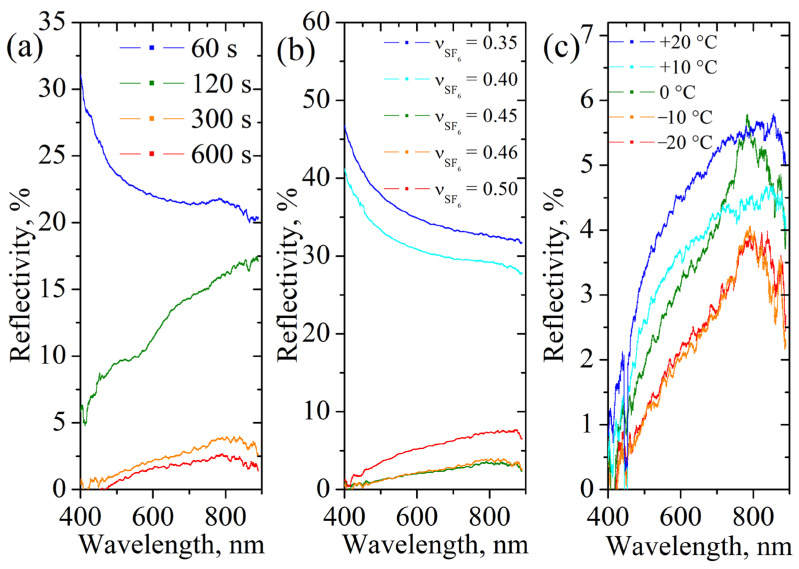
The spectral reflectivity coefficient of black silicon for different parameters of etching process: (**a**) etching duration (temperature is −20 °C, fraction of SF_6_ ($\nu_{{SF}_{6}}$) is 0.46); (**b**) SF_6_ fraction in plasma mixture (temperature is −20 °C, etching duration is 300 s); (**c**) wafer temperature (fraction of SF_6_ is 0.46, etching duration is 300 s).

In order to understand connection between topology and reflectivity, consider spectral reflectance measured for these samples using an integral sphere (Figure 8). Figure 8a shows the dependence of the spectral reflectance on the wavelength for different etching durations. It can be seen that when etched for 60 s, the reflection coefficient is approximately 20–30%, which is close to the values for smooth silicon. Increasing the etching duration to 120 s, at which, as was seen earlier, the continuity of the surface is lost, the reflection coefficient drops dramatically, and especially strongly in the short-wavelength region, the wavelengths in which are close to the autocorrelation length. With a further increase in the etching time, the magnitude of the reflection coefficient does not exceed 3%, and in the region less than 500 nm it does not differ significantly from the error.

Figure 8b shows the dependence of the reflection coefficient on the plasma composition at a constant etching time of 300 s. It can be seen that with a fraction of SF_6_ less than 0.4, in which the continuity of the surface (albeit with defects) is preserved, the values of the reflectance coefficient differ little from intact silicon. For samples with an SF_6_ fraction equal to 0.45–0.46, the minimum reflection coefficient does not exceed 3% over the entire measured spectrum, with a minimum at short waves. With a further increase in the proportion of SF_6_ (0.5) and the beginning of polishing etching, the reflection coefficient approaches 10%.

Separately, Figure 8c shows the spectral dependences of the reflectance on the temperature of the sample during etching. When the temperature changes from +20 °C to −20 °C, the etching depth decreases from 5.0 μm to 4.3, which is accompanied by a decrease in the autocorrelation length from 0.37 to 0. 28, while the reflection coefficient decreases monotonically, and there is no difference for temperatures −10 °C and −20 °C.

Preliminary calculations show that the combination of two dielectric layers with different refractive indices makes it possible to significantly reduce the reflection coefficient of the structure due to interference effects in the film. For the calculation, the matrix formalism [59] was used, which allows one to calculate the reflection coefficients of isotropic layered media. The refractive index and extinction coefficient of silicon in the optical range under study were taken from the literature [60]. And the refractive indices for HfO_2_ and Al_2_O_3_ were measured by spectral ellipsometry [52] on witness samples made of planar silicon. The extinction coefficients for HfO_2_ and Al_2_O_3_ were set to zero, which is true in this spectral range.

**Figure 9:**
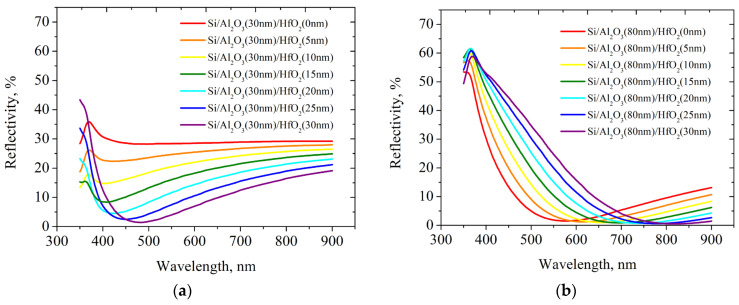
Modeled spectral reflectivity coefficient of plane two-layer antireflective coating: (**a**) Si(bulk)/Al_2_O_3_(30 nm)/HfO_2_(different thickness); (**b**) Si(bulk)/Al_2_O_3_(80 nm)/HfO_2_(different thickness).

Calculations were performed for two-layer films on planar silicon with a bottom layer of aluminum oxide with a thickness of 30 nm (Figure 9a) and 80 nm (Figure 9b). It should be noted that these calculations can only approximately characterize the system under study—they assume a smooth surface and normal incidence of the light beam. However, the presence of wide minima observed in the graph may mean that not only normally incident rays are effectively absorbed in this region of the spectrum.

Consider the qualitative difference between Figure 9a,b. In Figure 9a, as the hafnium oxide thickness increases, the reflection minimum becomes narrower but shifts slightly. In Figure 9b, which shows the results for a thicker aluminum oxide film, it is possible to achieve a shift in the region of maximum absorption, that is, there is a fundamental possibility by selecting film thicknesses to precisely tune the region of maximum absorption.

**Figure 10:**
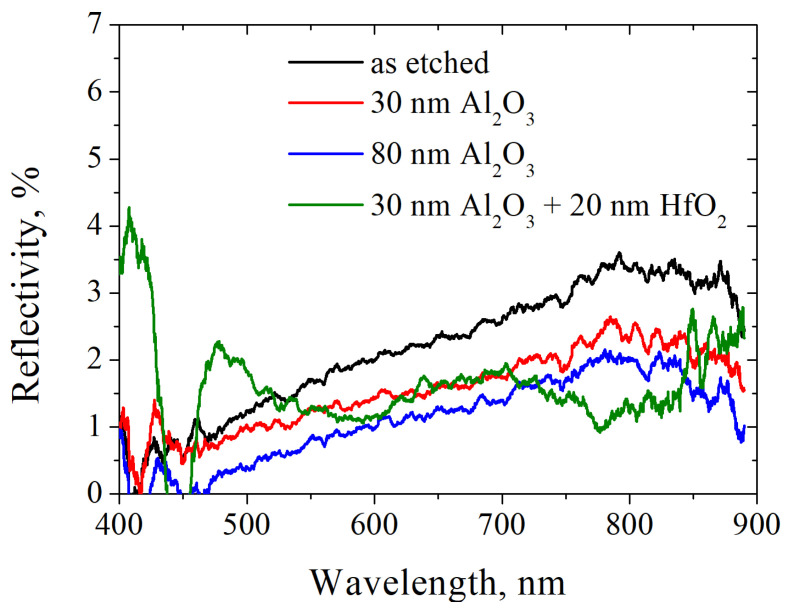
The measured spectral reflectivity coefficient of black silicon with different antireflective coatings. The black silicon etching parameters are as follows—temperature is −20 °C, fraction of SF_6_ is 0.46, etching duration is 300 s.

The experiments performed have shown that the use of a two-layer Al_2_O_3_/HfO_2_ coating makes it possible to reduce the reflection coefficient by up to 1.8 times (in different parts of the spectrum), see Figure 10.

Figure 10 shows a comparison of the measured reflectance of an uncoated and coated black silicon sample. It can be seen that the application of an aluminum oxide film monotonically reduces the reflection coefficient, and the use of a two-layer film makes it possible to obtain a sample with minimal reflection—no more than the measurement error (0.2%).

**Figure 11:**
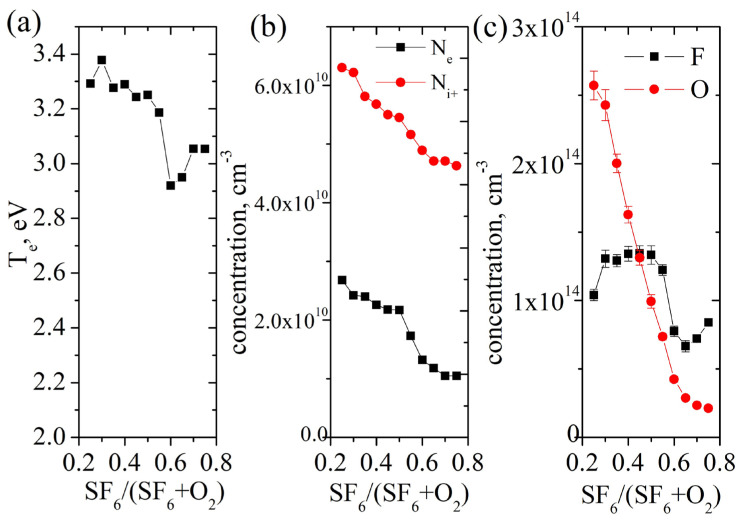
Plasma parameters over SF_6_ fraction in plasma mixture: (**a**) electron temperature; (**b**) electrons and positive ions concentrations; (**c**) fluorine and oxygen radical concentrations.

Let us consider the results of plasma diagnostics performed in the plasma etching tool. It was previously shown that there is no dependence of the plasma parameter on the temperature of the sample and the time of the etching process, which means that in our case we can neglect the flow of etching products into the reactor, which could contribute to chemical reactions and the equilibrium of particle concentrations in the plasma. It can be seen (Figure 11a) that the electron temperature measured (with a Langmuir probe) conservatively depends on the composition of the gas mixture, decreasing slightly and almost monotonically with increasing SF_6_ fraction. The concentrations of electrons and positive ions also decrease monotonically, but much more significantly (Figure 11b). The discrepancy in the concentrations of these particles is explained by the large fraction of negative ions, which obviously increases with increasing SF_6_ content. If at a fraction of SF_6_ equal to 0.25 approximately equal concentrations of electrons and negative ions are observed, then at a fraction equal to 0.75 the concentration of negative ions is 3.5 times higher than the concentration of electrons. These observations are in good agreement with the behavior of electronegative gases such as SF_6_ in low-temperature plasma with a low degree of ionization.

The behavior of the concentrations of fluorine and oxygen in the atomic form of radicals is shown in Figure 11c. It can be seen that the concentration of atomic fluorine generally follows the electron temperature graph, which explains the fluorine concentration by the balance between the birth of such particles due to the impact dissociation of molecular radicals SF_x_ and the death of fluorine on the walls. For atomic oxygen, the dependence is stronger and linear, decreasing as the proportion of SF_6_ increases.

## 4. Discussion

It was shown that the patterns of formation of black silicon depend mainly on the concentration of oxygen radicals on the composition of the plasma-forming mixture. As the proportion of SF_6_ in the mixture increases, the proportion of atomic oxygen in the plasma decreases, and the proportion of fluorine changes slightly. It was shown that the surface oxidation of silicon in plasma is mainly controlled by the concentration of atomic oxygen [61], and the etching rate of silicon and silicon oxide (in this case as a passivation layer) is determined by the concentration of atomic fluorine over a wide range of concentrations [62]. In addition, other factors, such as temperature and average ion energy, can shift the passivation-etching balance.

When the temperature changes from +20 °C to −20 °C, the etching depth decreases from 5.0 μm to 4.3, which is accompanied by a decrease in the autocorrelation length from 0.37 to 0.28, while the reflection coefficient decreases monotonically, and there is no difference for temperatures −10 °C and −20 °C. Mean reflectance coefficient calculated in range 350–800 nm falls from 3.1% (at +20 °C) to 1.28% (at −10 °C) without additional coatings.

It is especially important that the studied process is optimal even at temperatures of −10 °C, which are potentially achievable on conventional etching equipment in microelectronics using liquid chillers with antifreeze. This is important, since creating temperatures below −50 °C requires the use of liquid nitrogen, which significantly increases the cost of the process and, in addition, when loading a sample into the chamber through a load-lock, its temperature stabilization time increases, which significantly reduces productivity.

A novel approach has been studied to reduce the reflectance using conformal dielectric coatings deposited by atomic layer deposition. The reflectivity of the resulting black silicon was studied in a wide spectral range from 400–900 nm. We were able to offer an etching process that forms a black silicon surface with a range-average reflectance of 1.4%, and additional coating with two-layer coatings made it possible to reduce this figure to 0.8% (Al_2_O_3_—30 nm/HfO_2_—20 nm) and 0.5% (Al_2_O_3_—80 nm/HfO_2_—20 nm).

The possibility of changing the ACF by changing the etching duration and bias voltage has been established, which will make it possible to predictably change the spectral characteristics of reflection from the resulting material.

The approach with two-layer dielectric films makes it possible in principle to control the position of the reflection minimum in the spectrum, which can be used to obtain selective antireflection coatings.

## 5. Conclusions

The method for forming black silicon using plasma etching at a sample temperature range from −20 °C to +20 °C in a mixture of oxygen and sulfur hexafluoride was studied in depth. The surface morphology of the resulting structures, the autocorrelation function of surface features, and reflectivity were studied depending on the process parameters—the composition of the plasma mixture, temperature and other discharge parameters (radical concentrations).

As a result of the research, technologies for creating black silicon on silicon wafers with a diameter of 200 mm have been proposed, and the structure formation process takes no more than 5 min. The resulting structures are an example of the self-formation of nanostructures due to anisotropic plasma etching.

## Data Availability

The data that support the findings of this study are available from the corresponding author, A.M., upon reasonable request.
